# Elevated placenta growth factor predicts pneumonia in patients with chronic obstructive pulmonary disease under inhaled corticosteroids therapy

**DOI:** 10.1186/1471-2466-11-46

**Published:** 2011-09-30

**Authors:** Shih-Lung Cheng, Hao-Chien Wang, Shih-Jung Cheng, Chong-Jen Yu

**Affiliations:** 1 Department of Internal Medicine, Far Eastern Memorial Hospital, Pan-Chiao, Taipei, Taiwan; 2 Department of Chemical Engineering and Materials Science, Yuan-Ze University; Taoyuan, Taiwan; 3 Department of Internal Medicine, National Taiwan University Hospital, National Taiwan University, Taipei, Taiwan; 4 Division of Oral and Maxillofacial Surgery, Department of Dentistry, National Taiwan University Hospital, Taipei, Taiwan

**Keywords:** chronic obstructive pulmonary disease, placenta growth factor, inhaled corticosteroids

## Abstract

**Background:**

An increased incidence of pneumonia in patients with chronic obstructive pulmonary disease (COPD) under inhaled corticosteroid (ICS) therapy was noticed in previous studies. We performed a prospective study to elucidate the risk factors for the development of pneumonia in this group of patients.

**Methods:**

A prospective, non-randomized study with patients diagnosed as having COPD from 2007 to 2008 identified in the Far Eastern Memorial Hospital were recruited. We recorded data for all patients, including clinical features and signs, demographic data, lung function status, and medications. Bio-markers such as C-reactive protein (CRP) and placenta growth factor (PlGF) were checked at first diagnosis. Every acute exacerbation was also recorded, especially pneumonia events, which were confirmed by chest radiography. Multivariate analysis was performed with stepwise logistic regression for pneumonia risk factors.

**Results:**

274 patients were diagnosed as having COPD during the study period and 29 patients suffered from pneumonia with a prevalence of 10.6%. The rate was significantly higher in patients with ICS therapy (20/125, 16%) compared with those without ICS (9/149, 6%) (p = 0.02). We stratified ICS therapy into medium dose (500-999 ug/day fluticasone equivalent, 71 patients) and high dose (1000 ug/day and higher fluticasone equivalent, 54 patients) group. There was no statistical difference in the incidence of pneumonia between these two group (medium dose: 13/71, 18.3% vs. high dose: 7/54, 12.9%, p = 0.47). Multivariate analysis was performed to identify the risk factors for developing pneumonia and included forced expiratory volume in one second (FEV1) less than 40% of predicted (odds ratio (OR) 2.2, 95% confidence interval (CI): 1.1-6.9), ICS prescription ((OR) 2.4, 95% (CI): 1.3-8.7), the presence of diabetes mellitus (DM) (OR 2.6, 95% CI: 1.2-9.4) and PlGF level over 40 pg/L (OR 4.1, 95% CI: 1.5-9.9).

**Conclusion:**

ICS therapy in patients with COPD increased the risk of pneumonia. However, there was no relationship between the incidence of pneumonia and dosage of ICS. Additionally, advanced COPD status, DM and elevated PlGF level were independent risk factors for the development of pneumonia. PlGF would be a good novel biomarker for predicting pneumonia.

## Introduction

Chronic obstructive pulmonary disease (COPD) represents an increasing burden worldwide. It has been estimated that COPD will become the third most common cause of death globally by the year 2030 [1]. COPD is a disease characterized by progressive airflow limitation that is not fully reversible, with symptoms of dyspnea, sputum production and cough. The chronic airflow obstruction is associated with an abnormal inflammatory response of the lungs, and is caused primarily by cigarette smoking [2,3]. COPD is associated with significant morbidity and mortality and an increased risk of pneumonia [4,5]. Moreover, patients with COPD who are hospitalized due to pneumonia have been reported to be at higher risk of mortality than patients without COPD [6,7].

Several studies have demonstrated that inhaled corticosteroids (ICS) therapy can reduce the frequency of COPD exacerbations and improved health status [8-10]. The current Global Initiative for Chronic Obstructive Pulmonary Disease (GOLD) guidelines suggests ICS usage in symptomatic patients in severe or very severe stage and repeated exacerbations [11].

ICS is prescribed to as many as 50% of patients with COPD [12-14]. The TORCH (Towards a Revolution in COPD Health) study [15] reported a 19% 3-year rate of pneumonia in patients receiving fluticasone at 1000 μg/day, corresponding to a significant 1.6- fold increase over placebo. Additionally, several randomized controlled reports and meta-analysis studies have raised a possible safety issue regarding the higher rate of pneumonia in ICS treatment [16-19]. In contrast, other studies have reported different results in which an increased incidence of pneumonia was not seen with regard to ICS usage [20-23]. Therefore, whether or not the use of ICS is associated with an increased risk of pneumonia still needs to be elucidated.

Normally, PlGF mRNA is present most abundantly in the placenta, thyroid, and lungs [24], but the biologic function of PlGF in these tissues remains unclear. PlGF expression increases significantly in early gestation, peaks at around 26-30 weeks, and decreased as term approaches [25]. Recent studies have shown that PlGF can cause emphysema when over-expressed in the mouse lung [26], while knocking-out PlGF protected against the development of elastase-induced emphysema [27]. Cheng and colleagues [28] have also reported that PlGF is released from bronchial epithelial cells and potentially contributes to the development of COPD. Recently, a meta-analysis study showed that lower baseline lung function status was associated with an increased incidence of pneumonia development [29]. In addition, our previous studies revealed that serum placenta growth factor (PlGF) levels were inversely correlated to forced expiratory volume in one second (FEV1) in COPD, [28] and that PlGF was a good biomarker for predicting lung function and elevated levels in sepsis [30]. Additionally, C-reactive protein (CRP) has also been shown to be an important biomarker for systemic inflammation in COPD [31,32]. Therefore, we conducted a prospective study with the aim of assessing whether ICS usage in patients with COPD increased the rate of pneumonia, and whether PlGF and CRP could predict the development of pneumonia in patients with COPD using ICS.

## Methods

### Source of Data

This report utilized a prospective, non-randomized study from the Far Eastern Memorial Hospital. COPD was diagnosed on the basis of history, chest X-ray findings, physical examination, and spirometric data, according to the American Thoracic Society guidelines [11]. Inclusion criteria for COPD included: chronic airway symptoms and signs such as coughing, breathlessness, wheezing and chronic airway obstruction, which was defined as (1) FEV1/FVC less than 70% and (2) FEV1 less than 80% of the predicted value via spirometric data; and FEV1 reversibility after inhalation of 200 μg salbutamol of less than 12% of pre- bronchodilator FEV1. Subjects were excluded if they had a history of asthma (reversibility of airflow obstruction) or malignant lung disease. The follow-up time of the study was two years.

### Inhaled Corticosteroid Exposure

The ICS included inhaled beclomethasone, budesonide, and fluticasone, whether dispensed alone or in a combination inhaler with an inhaled beta-agonist. The prescription of ICS and other medications was determined by physicians according to patients' clinical status and lung function severity. The estimation of equivalence was used on the basis of relative topical potency and what experts consider to comparable doses according to the National Asthma Education and Prevention Program Expert Panel report 2 [23] and the Canadian asthma consensus statement [24]. Accordingly, the equivalent doses for inhaled corticosteroids were beclomethasone 100 μg, budesonide 80 μg and fluticasone 50 μg. All doses were converted to fluticasone equivalents and categorized according to defined daily doses of the most recent prescription as high (fluticasone 1000 μg/d or more), and medium (fluticasone, 500-999 μg/d) and no ICS use.

Acute exacerbation of COPD was defined when a patient presented with at least two of the following major symptoms (increased dyspnea, increased sputum purulence, increased sputum volume) or one major and one minor symptom (nasal discharge/congestion, wheeze, sore throat, cough) for at least two consecutive days [25,26]. All chest radiographs were assessed by the investigators (respiratory physicians). Pneumonia was confirmed when there was radiographic evidence of pulmonary infection (new infiltrates and consolidation on the chest roentgenography) and combined with the symptoms of acute exacerbation. If chest radiographs were not available, patients were excluded from our study.

### Collection of peripheral blood & quantification of serum biomarker levels

Peripheral blood from patients with COPD at initial diagnosis was collected in heparinized syringes and centrifuged within 15 minutes of collection. The plasma was kept at -70°C until analysis by a technician who was blinded to the patients' condition. The levels of PlGF in the serum were assayed by a standardized sandwich enzyme-linked immunosorbent assay (ELISA) method (R&D Systems, Minneapolis, MN) in duplicate according to the manufacturer's protocol. C-reactive protein (CRP) was also checked at the clinical laboratory department, Far Eastern Memorial Hospital. Each patients were checked these biomarkers in the baseline status after enrollment. Ethical approval from the hospital and informed consent from all study subjects was obtained.

Covariates included age, sex, and the severity of respiratory disease, as well as other conditions associated with a risk of pneumonia were recorded. We quantified the severity of respiratory disease, independently of inhaled corticosteroid use, by the number of dispensed prescriptions for respiratory medications (beta-agonists, tiotropium, theophylline). Another covariate was the number of antibiotic prescriptions in the past year, although excluding the last 30 days because such recent antibiotic prescriptions might have represented the initial therapy for the pneumonia that resulted in hospitalization.

Categorical variables were compared by the chi-square test, and continuous variables were compared by the Student's *t *test. A p value of less than 0.05 was considered statistically significant. To identify risk factors for pneumonia, multivariate analysis was performed with stepwise logistic regression to calculate the odds ratio. A Homer-Lemeshow goodnessof-fit test was used to help determine if the model was fit to the data well. All statistical analyses were performed using SPSS for Windows (SPSS 9.0 Statistical Package for Social Science, Inc., Chicago IL).

## Results

During the two-year study period, 274 patients were diagnosed as having COPD. 223 patients were males and 51 were females, with a mean age of 63.2 years old. Other demographic data such as lung function status, and smoking history are summarized in Table 1. The average baseline post-bronchodilator FEV1 was 46% of predicted. Among these patients with lung function assessment, 47 (17.2%), 118 (43.1%), 71(25.9%) and 38 (13.8%) patients had categorized as mild, moderate, severe, and very severe lung function, respectively, according to the GOLD classification [11].

**Table 1:** Demographic characteristics of the patients with COPD

Variables	COPD with ICS	COPD without ICS	*p *value
	(n = 125)	(n = 149)	
Age	66.7 ± 24.8	69.1 ± 25.1	0.62
Gender (M/F)	117/8	136/13	0.68
smoking (pack-years)	25.3 ± 14.2	22.1 ± 11.9	0.49
Lung function			
mild obstruction	0	47 (31.5%)	< 0.01*
moderate obstruction	41 (32.8%)	77 (51.7%)	0.04*
severe obstruction	54 (43.2%)	17 (11.4%)	< 0.01*
very severe obstruction	30 (24%)	8 (5.4%)	< 0.01*
Pneumonia	20 (16%)	9 (6%)	0.02*
C-reactive Protein (CRP) (mg/L)	4.8 ± 1.9	4.3 ± 2.2	0.45
Placenta Growth Factor (PlGF)(pg/L)	53.5 ± 18	37.6 ± 18.3	< 0.01*

According to the GOLD guidelines and clinical practice, ICS were prescribed for patients with severe and, very severe obstructive lung defects or frequent acute exacerbation (125 (45.6%) ICS users). Pneumonia was confirmed in 29 patients via chest roentgenography interpretated by a pulmonologists. The prevalence was 10.6%. The rate was significantly higher in patients with ICS therapy (20/125, 16%) compared with those without ICS therapy (9/149, 6%) (p = 0.02). We stratified ICS therapy into medium dose (500-999 ug/day fluticasone, 71 patients) and high dose (1000 ug/day fluticasone and more, 54 patients) two groups. There was no statistically difference in pneumonia development between these two group (medium dose: 13/71, 18.3% vs. high dose: 7/54, 12.9%, p = 0.47). (Table 2) Therefore, the risk of pneumonia development increased when using ICS therapy. There were four patients hospitalized for severe pneumonia. Among them, two patients were treated with higher dose of ICS; and the other two patients received medium dose of ICS therapy. There was no mortality due to pneumonia whether or not the patients were hospitalized.

**Table 2:** Demographic characteristics of the patients with COPD using inhaled corticosteroids

Variables	COPD with medium dose of ICS	COPD with high dose of ICS	*p *value
	(n = 71)	(n = 54)	
Age	64.1 ± 25.2	68.5 ± 24.4	0.36
Gender (M/F)	66/5	51/3	0.48
smoking (pack-years)	26.4 ± 13.6	25.1 ± 11.1	0.41
Lung function			
mild obstruction	0	0	
moderate obstruction	31 (43.7%)	10 (18.5%)	0.04*
severe obstruction	31 (43.7%)	23 (42.6%)	0.90
very severe obstruction	9 (18.3%)	21 (31.5%)	0.01*
Pneumonia	13 (18.3%)	7 (12.9%)	0.47
Hospitalization	2 (2.8%)	2 (3.7%)	0.78
C-reactive Protein (CRP) (mg/L)	4.8 ± 2.4	4.7 ± 2.7	0.62
Placenta Growth Factor (PlGF)(pg/L)	49.2 ± 23.5	51.3 ± 20.8	0.56

Serum biomarkers such as CRP and PlGF were also measured. There was no difference in the average levels of CRP in patients with ICS therapy (medium and high dose) (4.8 ± 1.9 mg/L) compared with those without ICS therapy (4.3 ± 2.2 mg/L, p = 0.45). However, PlGF levels were significantly higher in patients using ICS (53.5 ± 18.1 pg/L) than those without ICS (37.6 ± 18.3 mg/L, p < 0.01). (Table 1) Patients with ICS treatment had worse obstructive lung function in clinical practice and guidelines recommendations. In addition, there were no significant differences in the levels of CRP and PlGF between the COPD patients with the medium doses and those with high doses of ICS (Table 2).

Multivariate analysis was performed to identify the risk factors for developing pneumonia. After adjusting for sex, age, medications, and smoking status, significant factors including advanced COPD status (forced expiratory volume in one second (FEV1) less than 40% of predicted) (odds ratio (OR) 2.2, 95% confidence interval (CI): 1.1-6.9), ICS prescription (odds ratio (OR) 2.4, 95% confidence interval (CI): 1.3-8.7), the presence of diabetes mellitus (DM) (OR 2.6, 95% CI: 1.2-9.4) and PlGF level over 40 pg/L (OR 4.1, 95% CI: 1.5-9.9) (p < 0.01) were identified. (Table 3)

**Table 3:** Multi-variate analysis of risk factors for pneumonia in patients with chronic obstructive pulmonary disease.

Variables	No. of patients	No. of pneumonia (%)	Univariate analysis	Multivariate analysis
**Age**	128			
< 60 years	146	14 (10.9)	0.5 (0.1-5.7)	
≥ 60 years		15 (10.3)		
**Smoking **(pack-years)	157			
< 15	117	16 (10.2)	0.8 (0.5-4.3)	
≥ 15		13 (11.1)		
**Serum CRP **(mg/L)	163			
< 4.0	111	16 (9.8)	1.2 (0.6-6.1)	
≥ 4.0		13 (11.7)		
**Body mass index (BMI)**				
< 18	146	17 (11.6)	0.8 (0.4-6.7)	
≥ 18	128	12 (9.4)		
**COPD status**	75		2.5 (1.1-10.4)**	
Advanced (FEV1< 40%)	199	12 (16)		2.2 (1.1-6.9)**
Non-advanced (FEV1 ≥ 40%)		17 (8.5)		
**ICS prescription**	149		2.6 (1.4-8.9)*	2.4 (1.3-8.7)*
No	125	9 (6%)		
Yes		20 (16%)		
**Serum PlGF **(pg/L)	138		4.8 (1.3-11.2)*	4.1 (1.5-9.9)**
< 40	136	8 (5.8)		
≥ 40		21 (15.4)		
**DM**	127		2.3 (1.1-8.9)**	
No	147	11 (8.6)		2.6 (1.4-10.5)**
Yes		18 (12.2)		

*p < 0.05; **p < 0.01

CRP: C-reactive protein; FEV1: forced expiratory volume in one second; PlGF: placenta growth factor; DM: diabetes mellitus

All values are shown as mean ± standard deviation.

*Indicates a statistically significant difference between groups (p < 0.05).

## Discussion

The current guidelines for the management of COPD had recommended that ICS combined with long-acting β-agonist (LABA) could be prescribed for patients with severe disease and/or frequent exacerbations [11]. The effectiveness has been substantiated by recent publications demonstrating that ICS/LABA combinations result in a decrease frequency of acute exacerbations [8-10,15]. However, the present study found that ICS-containing regimens were associated with an increased risk of pneumonia, which confirmed the findings with other studies [15-19]. Therefore, this finding should raise the concern that ICS usage would increase the morbidity and hospitalization due to pneumonia in patients with COPD. Additionally, we found that there was no difference in the incidence of pneumonia between patients using medium or high doses of ICS treatment and a novel biomarker with PlGF can predict the development of pneumonia. Pneumonia development did not seem to be associated with dosage of ICS. The serum levels of PlGF seemed to reflect the severity of airflow obstruction and lung parenchymal inflammatory status in COPD patients.

The mechanism of an increased risk of pneumonia with ICS therapy is unknown. It is generally recognized that exacerbation of COPD are frequently associated with infection. Thus, there is a paradox in that ICS-containing regimens reduces the frequency of one infective complications of COPD (acute exacerbation), yet potentially increases another (pneumonia). It is possible that ICS can alter the local immune response and bacterial colonization in the airway, because ICS regimens are very effective in the management of the inflammation aspects of asthma [33]. However, there have not been any reports of an excess pneumonia incidence with ICS use in randomized clinical trials in asthma patients. Corticosteroids have been shown to be less effective in modulating neutrophil function (COPD) compared with other inflammatory cells such as eosinophils or mast cells (asthma) [33]. Some studies, however, had documented that ICS regimens still have the effect of reducing sputum neutrophilia in COPD patients [34,35], perhaps due to decreasing neutrophil chemotaxis [36].

In fact, clinicians still needs a good biomarker to reflect the actual inflammatory responses in the lung. PlGF is a 50-kDa glycosylated dimeric protein sharing significant sequence homology at the amino acid level with vascular endothelial growth factor (VEGF) [37]. Two isoforms of PlGF via alternative splicing of mature mRNA have been reported [38]. Like VEGF, PlGF exhibits mitogenic activity on cultured endothelial cells and induces angiogenesis *in vivo*, and its effects on endothelial cells are the same as to those of the potent classical angiogenic factors, such as VEGF and fibroblast growth factor [39]. We have confirmed that PlGF were increased in serum and BAL fluids of COPD patients, and correlated inversely with FEV_1 _[28]. In addition, bronchial cells can express PlGF via proinflammatory cytokines stimulation and over-expression of PlGF would induce apoptosis. It could be a novel biomarker to reflect the lung inflammation.

The symptoms of COPD acute exacerbations and pneumonia usually overlapped. This will cause the diagnosis difficulty especially without laboratory data and chest radiography. In this study, the diagnosis of pneumonia was in fact confirmed by radiographic finding. So, the data were more accuracy to elucidate the association between the ICS-containing regimens and pneumonia risk.

COPD itself is associated with an increased risk of pneumonia and the risk for pneumonia hospitalization is greater for patients with severe underlying disease. Additionally, higher dose of ICS were more likely to be prescribed to patients with more severe disease. To avoid confounding by severity lung function status, Ernst and collagens had reported that FEV1 severity were unlikely association with both likelihood of pneumonia or ICS prescription [40]. Several studies [15-19,41] were similar to our data that ICS regimen was the independent risk factor for an excess risk of pneumonia. Additionally, advanced COPD status (FEV1 less than 40% of predicted) was also an independent risk factor in our study. There seemed to be not correlation between two factors in statistical analysis. Clinicians should remain vigilant for the development of pneumonia with ICS therapy because the signs and symptoms of pneumonia closely mimic those of COPD exacerbation. Fortunately, all of these and our data had shown that any increase in pneumonia events with an ICS- containing COPD regimen dose not lead to increased pneumonia mortality.

By multivariate analysis, risk factors for pneumonia also included DM, and higher levels of PlGF (> 40 pg/dl). Hyperglycemia causes impairment in immune response and depresses neutrophil chemotaxis, phagocytosis, intracellular bacteriocidal activity, opsonization and cell-mediated immunity [42]. It is reasonable that patients with DM were relative in immunological dysfunction status, which was easily infected by microorganisms. Moreover, a recent data showed that COPD patients with DM co-morbidity had trend to have prolonged length of hospital stay and increases risk of death in acute exacerbation [43].

Elevated PlGF expression had harmful effects in lung epithelial cells and inversely correlated to lung function status in patients with COPD [28]. The expression of PlGF in COPD results from the stimulation of pro-inflammatory mediators such as tumor necrosis factor-α (TNF-α), Interleukin-8. Therefore, higher PlGF levels imply more inflammatory processes in the lung parenchyma, which prone to acute exacerbations or the development of pneumonia. It should be addressed that PlGF expression was not only associated with lung function severity but also an independent risk factor for predicting pneumonia development. Besides, we also found that PlGF levels were more sensitive in the prediction the pneumonia (or airway inflammation) than CRP. CRP has been shown to be a reliable biomarker for COPD in systemic inflammation responses and cardiovascular events [44], however, it is not good marker for predicting pneumonia in COPD patients with ICS treatment. Further studies should be performed and to elucidate whether PlGF expression can reflect the systemic inflammation response in COPD.

Our study is limited by its non-randomized study and relatively small sample size. So, we could not determine that the dose dependence of ICS was associated with the risk of pneumonia development. Further prospective, randomized studies with larger populations and more laboratory investigations should be conducted to elucidate the mechanism for ICS treatment and pneumonia in COPD patients and more reliable bio-markers to predict the important issue.

## Conclusions

In conclusion, ICS therapy in patients with COPD increased the risk of pneumonia, which was not dependent on the doses of ICS. Clinicians should closely monitor the clinical symptoms and signs in COPD patients with ICS therapy to detect the development of pneumonia and administer antibiotics treatment in a timely manner. Additionally, advanced COPD status, DM and elevated PlGF level were independent risk factors for pneumonia development. PlGF was a novel bio-marker to predict the pneumonia status for COPD patients with ICS therapy.

## Competing interests

The authors declare that they have no competing interests.

## Authors' contributions

SLC carried out study design and performance and drafted the manuscript.

HCW conceived of the study, and participated in its design and coordination. SJC carried out ELISA. CJY conceived of the study, and participated in its design and coordination. All authors read and approved the final manuscript.

## Pre-publication history

The pre-publication history for this paper can be accessed here:

http://www.biomedcentral.com/1471-2466/11/46/prepub
